# Predicting the Impact of Climate Change on the Selection of Reintroduction Sites for the South China Tiger (*Panthera tigris amoyensis*) in China

**DOI:** 10.3390/ani14172477

**Published:** 2024-08-26

**Authors:** Yueqing Luo, Jin Xu, Xinyi Zhang, Yulin Hou

**Affiliations:** 1 School of Architecture and Urban Planning, Guangzhou University, Guangzhou 510006, China; 2112209056@e.gzhu.edu.cn (Y.L.); 2112209047@e.gzhu.edu.cn (X.Z.); 2112209041@e.gzhu.edu.cn (Y.H.); 2 National Park Research Center, Guangzhou University, Guangzhou 510006, China

**Keywords:** South China tiger, reintroduction, future climate, biodiversity, prey, MaxEnt model, suitable habitats

## Abstract

**Simple Summary:**

The South China tiger is a unique subspecies of tiger endemic to China and has become extinct in the wild. However, it is currently unclear whether there are still ideal habitats suitable for the survival of the South China tiger population in China at present and in the future. Our goal is to assess which areas will become the most suitable for the reintroduction of the South China tiger to China under the impact of climate change, and to estimate the number of tigers these areas can support. This study selects eight key prey species of the South China tiger, and predicts the potential suitable habitats for each prey under current and future climates. The study reveals that the core candidate sites for the reintroduction of the South China tiger cover a total area of 83,415 km^2^, with the largest core candidate site area totaling 10,000 km^2^, located in Tibet, which can ideally support the survival of 89 South China tigers in the wild. This study provides a research basis and strategy for the recovery of wild South China tiger populations.

**Abstract:**

The South China tiger (*Panthera tigris amoyensis*) is a tiger subspecies unique to China and one of the top ten endangered species in the world. It used to play an important role in the overall function of the ecosystem. This study rationally screened out key prey species of the South China tiger—the Chinese serow, Chinese goral, tufted deer, water deer, Chinese muntjac, red muntjac, sambar deer, and wild boar. Candidate sites for the rewilding and reintroduction of the South China tiger were derived by exploring changes in suitable habitats for the prey using the MaxEnt model. The results show that: (1) by 2070, except for the high-suitability areas of water deer and Chinese muntjac, the areas of suitable habitats for the other six prey species would all have decreased significantly; (2) the location of the high-suitability area of the South China tiger obtained by superimposing the suitable areas of the eight prey species would be almost stable in 2050 and 2070, but the habitat index of some high- and medium-suitability areas would decrease and turn into low-suitability areas; (3) the core candidate sites were 83,415 km^2^ in total, of which 25,630 km^2^ overlapped with existing protected areas, accounting for 30.7% of the core candidate sites, and the remaining 69.3% of the core candidate sites were mostly distributed around the protected areas; (4) the maximum core candidate site area was projected to be 10,000 km^2^ by 2070, which could support a small population of 23 male tigers and 66 female tigers to survive and reproduce in the wild. This study revealed the core candidate sites for the rewilding of South China tigers and estimated the number of tigers that could be reintroduced to these areas, providing a preliminary research basis for promoting the rewilding of South China tigers in China.

## 1. Introduction

Habitat loss, fragmentation, and degradation are prominent issues facing global biodiversity [1,2]. Climate change can alter the range and abundance of species’ habitats. Due to predicted global warming, the changes in suitable habitats for many species will be further exacerbated [3,4,5]. For example, in future climate scenarios, global warming and precipitation changes will cause species to migrate northward, which will cause many species to lose large areas of their original habitats or migrate to high latitudes [6,7,8]. Therefore, studying the changes in species’ habitats under different carbon emission scenarios at different times will help us understand the degree of adaptability of species to climate change, which is crucial to biodiversity conservation strategies. At the same time, human activities are considered to be the main cause of habitat loss and fragmentation, and have also had direct and indirect impacts on species migration and range [9,10]. At present, the Human Footprint Index has been widely used to analyze the impact of human activities on wildlife and habitats [11]. Therefore, it is crucial to consider the negative impact of the human footprint on wildlife, while considering climate change in order to determine the priority protection areas for specific species.

Large carnivores play an important role in maintaining the stability of ecosystems [12]. Tigers are one of the most endangered large carnivores in the world, and the South China tiger (*Panthera tigris amoyensis*) is the most endangered of all tiger subspecies and one of the top ten endangered species in the world [13]. As a flagship species at the top of the ecological chain, the South China tiger used to play an important role in maintaining the balance and improving the overall function of forest ecosystems. However, there have been no traces of South China tigers in the wild since 1991. The main factors for the extinction of the wild South China tiger were hunting, reduction of prey, and habitat destruction [14]. In recent years, with the strengthening of law enforcement for wildlife protection in China and the establishment of the nature reserve system, illegal hunting and trafficking of wildlife and plant products have been effectively curbed [15]. The populations of ungulate animals, which are the main food sources for predators, have also seen significant growth [16]. Therefore, a vast, ecologically diverse, and food-rich habitat is a prerequisite for the success of its reintroduction at this stage. The abundance and accessibility of prey have been identified as key factors affecting the survival and activity patterns of wild tigers [17]. There is a high correlation between tigers and prey activity patterns [18,19]. Given that the area where the prey species survive is a key factor in the continued survival of the South China tiger [17,18,20], this study determined that the prediction of suitable areas for the South China tiger’s key prey habitats can serve as a reasonable alternative to the prediction of potential suitable areas for the rewilding of the South China tiger.

The study concludes that China has the largest area of effective potential habitat (513,572 km^2^) among all tiger distribution countries and is therefore considered one of the countries with the most effective potential tiger habitat [21]. It is more urgent to quantify the potential habitat for the reintroduction and sustainable survival of the South China tiger population in China. According to Qin’s survey of 68 experts, more and more international experts support the reintroduction of South China tigers [22]. Since the 21st century, the Chinese government has actively promoted the reintroduction of South China tigers into the wild. In 2002, the South China tiger reintroduction project was approved and implemented, and in 2010, South China tigers were included in the “China Wild Tiger Population Recovery Plan” [23]. The Chinese government is actively looking for areas suitable for the reintroduction of South China tigers and has been continuously promoting the South China tiger breeding base to strengthen habitat transformation and restoration, and South China tiger rewilding training [24].

According to the minimum habitat area of 1000 km^2^ proposed by tiger experts for restoring the wild population of South China tigers [25], Hupingshan-Houhe National Nature Reserve is considered to be the largest and most suitable area for the reintroduction of South China tigers in China [14]. Although experts have analyzed the priority introduction sites of South China tigers in the reserve and evaluated their reintroduction potential [26,27,28], the total area of the reserve is 1100 km^2^, the available habitat is fragmented, and it can at most support a small population of the species with two to nine South China tigers [26]. Therefore, in the long run, the restoration of the South China tiger population in China can only be achieved when more, larger, and more stable habitats are found to support larger prey populations. Since 2000, China’s ecosystems have been gradually restored and the quality of biological habitats has been continuously improved [29]. Whether there is still an ideal habitat suitable for the survival of South China tiger populations now and in the future is a question that needs to be answered.

To fill this research gap, this study used the MaxEnt model to predict the potential suitable habitats of various prey species in combination with current and future climates. We explored: (1) the changes that will likely occur in the suitable habitats of prey species under the influence of the future climate; (2) the changes that will likely occur in the areas of wild release of South China tigers due to the influence of future climate on the suitable habitats of prey species; (3) what the relationship is between the relatively stable areas of South China tigers, China’s existing protected areas, and the human footprint; (4) how many South China tiger populations the core candidate areas can support to survive and reproduce.

## 2. Materials and Methods

### 2.1. Data Sources and Processing

#### 2.1.1. Prey Species Screening

The main prey of the South China tiger are Artiodactyls, such as wild boar, red muntjac, and sambar deer [30], and it prefers medium and large prey [17,20]. According to the experts’ suggestions, this study screened the main prey of the South China tiger in the following steps: (1) the initial prey data were screened from the Artiodactyls under the Chinese species list on the Species 2000 China Node website (http://www.sp2000.org.cn/, accessed on 8 May 2023), and a total of 46 species were obtained (Table S1); (2) according to the prey preference of the South China tiger, Bovidae, Camelidae, Cervidae, Musk Deer, and Suidae species were screened, and a total of 45 species were obtained [31,32]; (3) then, 14 species were obtained based on the historical distribution area of the species, which was south of the Qinling-Huaihe line [30]; (4) finally, according to the species protection status, the national first-class protected wild animals of China were excluded, and a total of 8 key prey species were obtained (Table 1).

#### 2.1.2. Species Distribution Data Search and Screening

**Figure 1:**
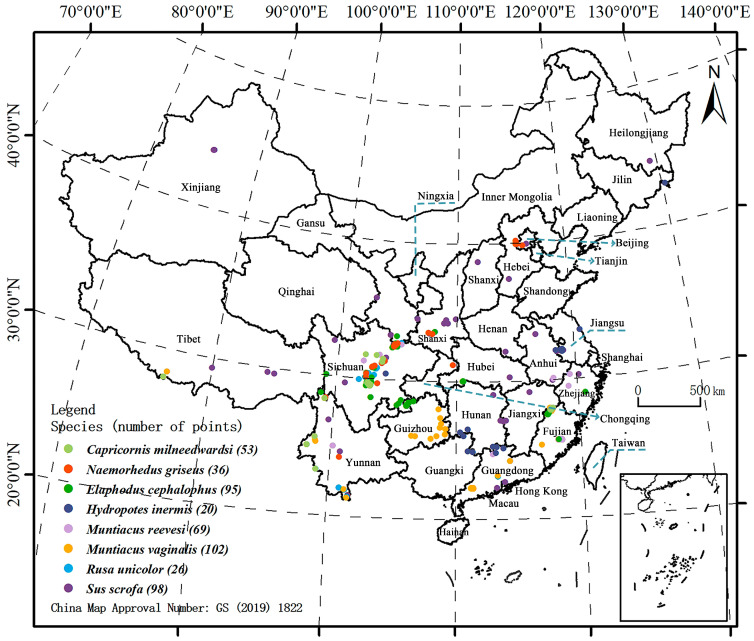
Distribution of selected sites of eight key prey species in China.

The geographical distribution point data of the eight key prey species of the South China tiger were obtained from the Global Species Diversity Information Base (https://www.gbif.org/, accessed on 21 May 2023) and related literature, and information from a total of 747 distribution points in China was collected. In order to remove the correlation between the distribution points, the SDM Toolbox tool in ArcGIS 10.8 was used to screen the eight prey-species distribution points that were collected. Ultimately, 499 distribution points for prey species were selected for model simulation (Figure 1).

#### 2.1.3. Environmental Variable Data Sources and Processing

Based on the principles of scientificity and representativeness, this study selected environmental factors that had been proven to be important for the habitat of ungulate species [33,34,35] as candidate factors related to the habitat of prey species of South China tigers, including 4 categories of climatic factors, topographic factors, water source vegetation factors, and human disturbance factors, with a total of 27 environmental factors (Table 2). The 27 environmental variables were clipped according to the administrative regions of China, with the co-ordinate system unified and the spatial resolution resampled. Thereafter, the processed raster data were uniformly converted into ASCII format [36]. Finally, the correlation tool in the ENMTools 1.4.4 software was used for correlation analysis, and the factors with a correlation coefficient (|r| ≥ 0.8) and a low contribution rate to the model were removed to obtain the environmental variables retained by each prey species (Figure S1).

This study used climate scenario data for the present (1970–2000), 2050 (average of 2041–2060), and 2070 (average of 2061–2080). These include four shared socioeconomic pathway scenarios: SSP1—2.6 (representing a low forcing scenario), SSP2—4.5 (representing a moderate forcing scenario), SSP3—7.0 (representing a high forcing scenario), and SSP5-8.5 (representing an extremely high forcing scenario). In order to avoid the uncertainty of different SSPs in predicting the future climate, this study selected SSP1—2.6, SSP2—4.5, SSP3—7.0, and SSP5—8.5 to predict the average suitable distribution area under the climate conditions of 2050 and 2070.

### 2.2. Model Optimization and Construction

The default parameter results of the MaxEnt 3.4.4 model were considered unreliable [37] and were prone to overfitting the sampled data, so this study used the Kuenm package in R 3.6.3 to optimize the MaxEnt model parameters [38,39]. The distribution point data of the eight prey species and the screened environmental variables were entered into the MaxEnt model for calculation. Using the parameters optimized by the regulation ratio and feature combination, 75% of the distribution point data were randomly selected for simulation training, and the remaining 25% of the point data were used as test data [37].

The MaxEnt model used the AUC (Area Under Curve) value under the ROC (Receiver Operating Characteristic) curve to test the accuracy of the results [40]. The AUC value ranged from 0 to 1. The larger the value, the higher the model accuracy, indicating that the environmental variables were more closely related to the geographical distribution of species [41]. When the AUC value was between 0.5 and 0.6, it was unqualified; between 0.6 and 0.7, it was poor; between 0.7 and 0.8, it was fair; between 0.8 and 0.9, it was good; between 0.9 and 1.0, it was excellent [42].

### 2.3. Drawing of Distribution Map of Prey and Habitat Suitable for South China Tiger

The MaxEnt model calculated a 0–1 logical index of suitability as the average of 10 operations. The higher the value, the higher the habitat suitability index of the species. We performed spatial overlay analysis and standardized the suitable areas of all prey with the same weight to obtain the potential suitable area distribution of the South China tiger in China. In order to select the effective long-term protected habitat area of the species from the current and future prediction maps, we superimposed the current, 2050, and 2070 habitats of the South China tiger to obtain a relatively stable wild release area for the South China tiger. ArcGIS was used to visualize the model results, and all the suitable area results were divided into four levels: high, medium, low, and non-suitability areas. Finally, ArcGIS software was used to calculate the grid area to obtain the area of the species in each level of the suitable area, and a distribution map of the suitable area level was drawn. In order to evaluate the changes in the species’ habitat, we quantified the changes in the habitat by comparing the current distribution map with the future distribution map, thereby showing the impact of future climate on prey and South China tigers.

### 2.4. Discussion of the Protection Status of Core Alternative Sites

We extracted the highly suitable habitats of the South China tiger after superposition and screened out patches of less than 100 km^2^, which were considered preliminary candidate sites for the rewilding of the South China tiger. In order to screen out the impact of human footprints on the release sites of the South China tiger, we used the 2020 global human footprint [43] to superimpose and compare the protected areas where tigers are currently known to survive in India, China, Bhutan, and other countries with human footprints. By calculation, it was found that the human footprint index within the long-term stable survival range of tigers was less than 8.0. Based on this result, preliminary candidate sites with a human footprint index higher than 8.0 and accounting for more than 50% of the patch area were also screened out, and the core candidate sites for the rewilding of the South China tiger were obtained. Given that the species richness in protected areas in China is higher than that outside protected areas [21], we believe that protected areas are more suitable for the survival of South China tigers in the wild. We used the boundary vector data of 1028 nature reserves across the country, released in 2021 by China’s ten national parks and the China Nature Reserve Specimen Resource Sharing Platform, overlaid and compared the core candidate sites with protected areas, explored the protection status of the core candidate sites, and determined the priority protected habitats for the rewilding and release of the South China tiger.

### 2.5. Estimation of Carrying Capacity of Core Candidate Sites

Suitable habitats for the South China tiger were previously obtained by superimposing the suitable habitats of prey, then removing areas with high human footprint. The prey resources in the core habitat were assumed to be stable and sufficient, with less human impact. This estimated that the core candidate site could support the number of South China tigers. According to Table S2, the estimated value of the highest density was excluded, and then the average density of tigers was calculated. Although the average value was more reasonable, for the sake of conservatism, the middle value between the average value and the minimum value was finally taken, that is, 0.46/100 km^2^ for males and 1.32/100 km^2^ for females, and this was used as a basis to calculate the carrying capacity of the core candidate site without superimposing the ranges of male and female tigers.

## 3. Results

### 3.1. Changes in Suitable Habitats for Prey under Climate Change

From the geographical distribution of suitable habitats (Table 3), only the areas of highly suitable habitats of red muntjac and sambar expanded, indicating that both were more adaptable to the trend of global warming, and it was speculated that they had a great correlation with their preference for warm and humid climates. Red muntjac expanded around the original highly suitable habitat, while sambar expanded to the northwest. The area of highly suitable habitats increased by an average of 130% in 2050 and 126% in 2070. The area of highly suitable habitats of tufted deer will first expand by 11% in 2050, then shrink by 64% in 2070. It is possible that short-term climate change favors the expansion of tufted deer, but has the opposite effect beyond its adaptive range. The areas of highly suitable habitats for the remaining five prey species all showed a shrinking trend. In 2050, the shrinking trend of Chinese goral and Chinese muntjac was the most obvious, shrinking by 54% and 87%, respectively. The shrinking trend of Chinese serow, water deer, and wild boar was relatively small, none exceeding 10%. By 2070, the areas of highly suitable habitats of the five prey species shrank significantly, shrinking by an average of 59%. It was worth noting that the movement of the highly suitable habitats of most prey species did not change much, with only water deer and sambar moving to higher latitudes (Figure S2).

### 3.2. Distribution and Changes of the Reintroduction Area of the South China Tiger under Climate Change

**Figure 2:**
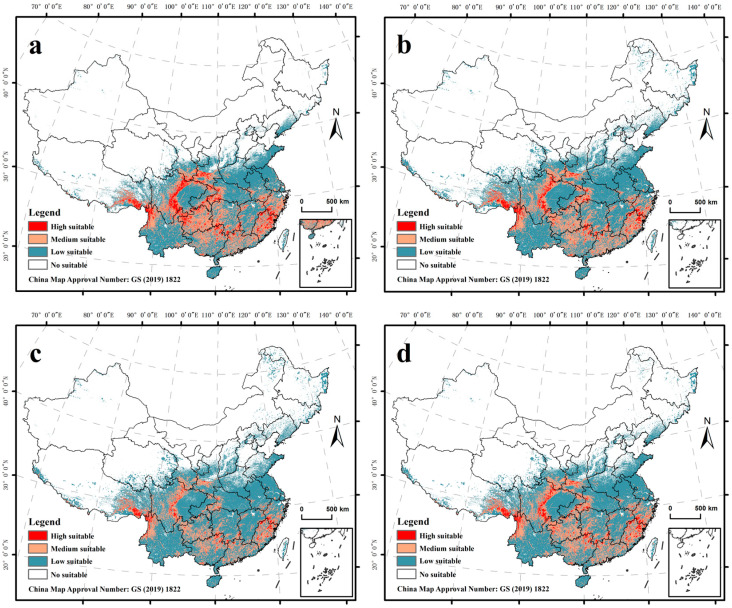
Prediction of the distribution of potential habitats of South China tigers. (**a**) Current habitat distribution of South China tigers; (**b**) habitat distribution of South China tigers in 2050; (**c**) habitat distribution of South China tigers in 2070; (**d**) the relatively stable habitat distribution of South China tigers after superposition.

This study used the superposition space of all prey habitats as the potential suitable habitat of the South China tiger in China. Given the impact of climate change on suitable habitats for prey, the potentially suitable habitat of the South China tiger also underwent corresponding changes in scope. From the prediction map, it could be seen that the potentially highly suitable habitat of the South China tiger was relatively continuous in the areas around the Sichuan Basin, and was fragmented in Fujian, Hunan, Guangxi, Guizhou, and other places. The medium-suitable habitat was mainly concentrated in the central and southern Guangdong Province, western Hunan Province, eastern Guizhou Province, and other areas. The low-suitable habitat was mainly concentrated in central and southern Yunnan Province, northern Anhui Province, central and southern Henan Province, and other areas, with the rest of the area being non-suitable habitat (Figure 2).

The results of the study found that the area of highly and medium-suitable habitats of the South China tiger will shrink by 29% and 6%, respectively, in 2050, and the area of low-suitable habitats will expand by 23%. By 2070, the area of highly and medium-suitable habitats will have shrunk by 64% and 26%, respectively, and the area of low-suitable habitats will expand by 30% (Table 3). The changes within each suitable habitat in 2050 and 2070 will specifically manifest in the reduction of the area of highly and medium-suitable habitats, and the increase in the area of low-suitable habitats, indicating that under the influence of the future climate some highly and medium-suitable habitats would reduce the habitat index and turn into low-suitable habitats by 2050 and 2070. Although the potential distribution of the South China tiger in the future was very similar to the current potential distribution, the results showed that under the influence of the future climate, the suitable habitat of the South China tiger will shrink significantly, which also means that the trend of global warming will adversely affect the release of the South China tiger into the wild.

### 3.3. Identify Core Candidate Sites for Priority Protection

**Figure 3:**
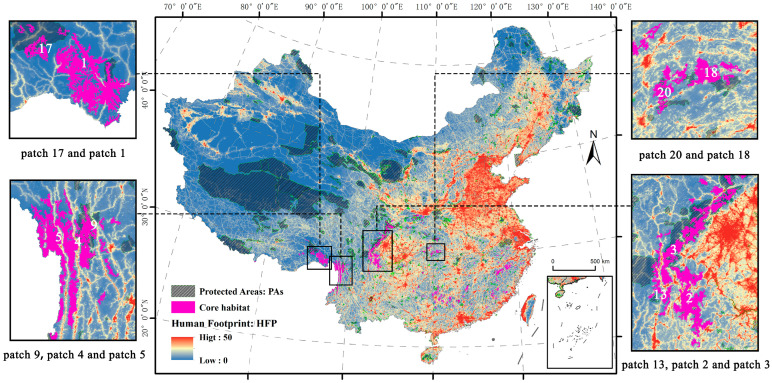
Relationship between core candidate sites, human footprints, and protected areas.

This study found 141 patches in the core candidate sites, totaling 83,415 km^2^, of which 25,630 km^2^ (31%) were in protected areas (Figure 3). From Figure 3, it could be seen that, although 69% of the core candidate sites were not in protected areas, these habitats were mostly distributed around protected areas, indicating that most of the core candidate sites were affected by protected areas and showed a low human footprint index. We ranked the top 20 core candidate sites by their area (Table 4) and found that 17 patches were larger than 1000 km^2^. Additionally, seven patches were not covered by protected areas, but four of them were around protected areas. We estimated that the remaining three patches might have been wilderness with high habitat quality. Furthermore, we identified that some patches were close to each other, such as patch 13, patch 2, and patch 3; patch 1 and patch 17; and patch 18 and patch 20. Notably, by superimposing them with human footprints, we found these patches were adjacent, but not connected by human footprints, and were isolated by roads and railways.

### 3.4. Core Candidate Sites Can Support the Survival of Small- and Medium-Sized Populations of South China Tigers

Under the condition of ideal prey quantity and low human disturbance, we estimated the number of South China tigers that the top 20 patches could support (Table 4). We found the current large patches in China (10,054 km^2^) could support 23 male tigers and 66 female tigers under ideal conditions. When the patch met the minimum release target area of about 1000 km^2^, it could support a small population of two male tigers and six female tigers under ideal conditions. The results showed that, under the influence of the future climate, the survival population of South China tigers was still considerable.

## 4. Discussion

### 4.1. Selection of Prey 

Because the South China tiger was extinct in the wild, there were no site data to infer habitat selection preferences, so this study inferred through prey. The lack of ungulate prey was a key factor limiting tiger recovery and was related to the survival and reproduction of wild South China tiger populations [44]. Previous studies had shown that it was reasonable to analyze the habitat of the South China tiger through prey [26], and studies had confirmed that tigers gave priority to medium and large prey when hunting, and only turned to smaller prey when these prey species did not exist or were extremely low in number [45]. Previous scholars had only studied wild boars and sika deer [26], or single wild boars [27,28]. As wild boars are large, aggressive, and difficult to kill by semi-wild South China tigers, only focusing on wild boars or prey dominated by wild boars would be inappropriate, and the prey of the South China tiger soon after release must be met as comprehensively as possible. This study comprehensively considered the hunting behavior of South China tigers in different situations and selected the prey that was most suitable for the diet of South China tigers step-by-step, based on prey type, activity range, body size, and protection status. Finally, eight prey species weighing 9–200 kg were reasonably selected, which could more comprehensively meet the feeding requirements of South China tigers in the early stage of wild release compared with current research.

### 4.2. Impacts of Climate Change on Prey and the South China Tiger

Overall, under the influence of climate change, by 2070, although the eight prey species studied in this study did not become extinct, their suitable habitats were greatly reduced, and red muntjac and sambar will clearly move to higher latitudes. Similar results were obtained in other studies, such as the impact of climate change on the distribution of ungulate species on the Tibetan Plateau and the impact of climate change on leopard habitat [5,33,46]. The impact of global warming on species that tended to move to high latitudes was widely confirmed [47,48]. Given the impact of climate change on prey habitat selection, if the South China tiger remains in its original area, it will inevitably face the crisis of reduced prey and increased difficulty in hunting. For example, studies have shown that tigers choose prey according to the prey conditions in the environment. If there was no suitable ungulate prey in the environment, they turned to preying on livestock [49,50]. This will greatly increase the conflict between the South China tiger and the residents of the surrounding villages. Of course, it is more likely that the South China tiger will move to areas rich in ungulate species simultaneously with the distribution of prey. This study was also based on this hypothesis of being able to infer the suitable distribution range of the South China tiger under the future climate. Therefore, only by restoring the number and habitat of more ungulate prey populations could conditions be suitable for tiger recovery, and the promotion and spread of prey and tiger populations [51,52].

### 4.3. The Reintroduction of the South China Tiger into the Wild Requires a Larger and More Stable Habitat

The past research on South China tigers was concentrated in the Hupingshan-Houhe Nature Reserve and the Meihuashan National Nature Reserve [26,27,28,53]. There are more areas where South China tigers have historically lived and other suitable areas that may emerge in the future that are worth studying and exploring. Therefore, we believe that the demand for the wild release of South China tigers should not be limited to one area because China’s current habitat area is, indeed, difficult to meet the survival of large populations of South China tigers. We should make full use of large core candidate sites and give priority to small populations with multiple distributions. This result also reveals other areas besides the protected areas where South China tigers have historically lived, such as the core habitat on the border between Yunnan and Tibet. It is worth noting that this part of the core habitat is adjacent to India and Myanmar, which means that future transnational protection will be one of the important measures to restore the South China tiger population, requiring comprehensive co-operation among countries, and advance planning and deployment.

According to the results of this study, 17 core candidate sites with patches larger than 1000 km^2^ have been identified. These habitats can meet the survival and reproduction needs of small- and medium-sized populations of South China tigers. Existing studies have shown that tiger protection efforts should be extended beyond protected areas, and that ecological corridor networks play an important role in the distribution of tigers, facilitating communication and diffusion between populations [54,55,56]. The success of tiger reintroduction does not entirely depend on the source of the tiger, but more on the conditions of the reintroduction site. It is easier to succeed in areas with low human disturbance, high prey density, and a conservation management system [57,58]. However, most of the habitats are located outside the existing protected areas, and there are many habitat patches that are small or adjacent, which affects the spread of the South China tiger population. This means that if multiple release sites that meet the minimum area are to be established, the scope of the protected area must be expanded, or corridors must be constructed to connect adjacent protected areas [56,59,60]. Therefore, we recommend that habitats located outside the protected areas be included in the ecological protection red line areas for strict protection. Conservation projects should be initiated by environmental organizations such as The Nature Conservancy (TNC) and the China Ecological Environmental Protection Association (CEEPA). These projects should strengthen the monitoring and supervision of habitats outside the protected areas, as well as the management of human activities. At the same time, raising the ecological conservation awareness among local residents and encouraging their participation in ecological protection is essential.

Humans have a great impact on wildlife. Studies have shown that roads, human activities, and poaching are the main reasons for the decline and extinction of tigers [61,62,63]. The current road network may reduce the number of tigers and prey by more than 20%, and roads will become a common challenge for tiger recovery in the future [64]. In order to stop poaching and prevent conflicts between humans and wildlife, many tiger-surviving countries have taken various measures to prevent human–tiger conflicts. For example, India has adopted a real-time alarm monitoring system with cameras [65]. At the same time, poaching of wildlife has been widely curbed in China. Therefore, the release site of the South China tiger should be selected in an area far away from the village and should be far away from the road, or an ecological corridor with sufficient width and habitat conditions should be constructed across the road [66,67]. At the same time, the management department should strengthen the management and monitoring of South China tigers after their release, such as through tracking population stability, fecal health, occurrence sites, prey species, and abundance, etc.

### 4.4. Assurance for Reintroduction Projects

Studies have demonstrated that South China tigers born in captivity can successfully hunt moving prey freely, making them a viable source for populations intended for reintroduction into the wild [68]. Current reports indicate that the number of South China tigers kept in captivity in China has exceeded 240, with 20 in the Meihua Mountain area of Fujian already capable of hunting large live prey such as wild boar, and meeting the tiger experts’ recommendation of a minimum of 15–20 individuals per population [25]. Although all captive South China tigers are descendants of six individuals captured from the wild, research shows that the existing captive population still maintains a moderate level of genetic diversity, indicating that the South China tiger has good potential for population recovery [69]. Our research concludes that the largest core potential site could ideally support a population of 89 South China tigers, but, inevitably, small populations are more susceptible to the risk of extinction due to an inability to maintain genetic diversity [70]. Therefore, to enhance the genetic diversity of wild South China tiger populations, we recommend the following: (1) establish a germplasm resource bank for the South China tiger and construct a genomic map to more comprehensively preserve and record the genetic information of the species; (2) continue to strengthen the breeding work of captive South China tigers, and select high-quality individuals with significant genetic differences as candidates for reintroduction; (3) regularly monitor the genetic health of the reintroduced South China tiger populations, and assess genetic diversity and adaptability.

## 5. Conclusions

Because this study could not obtain comprehensive data on prey population density, it did not consider the impact of prey population density on the distribution of the South China tiger’s suitable habitat, and the method for estimating survival potential was also quite approximate. However, the latitude and longitude information points of the species we collected were all locations where the prey was photographed more frequently, so the estimation of prey population density can be assumed to have been conducted to a certain extent. In addition, the population of wild boar in many areas is too high at present. Both red muntjac and Chinese muntjac are year-round breeding species of no concern, and their populations have increased [16]. If future research conditions are sufficient, this environmental factor can be taken into account. At the same time, in the operation of the MaxEnt model, although the sample size of the species in this study met the requirements, the distribution data of some samples were concentrated in a specific nature reserve, which will have a certain impact on the model prediction results. If future research conditions are sufficient, more and more comprehensive species distribution point data can be collected to obtain a more accurate prediction range of potentially suitable habitats.

In future research, field surveys should be conducted on the selected core candidate sites to determine the number of prey, population density, and human disturbance in the candidate sites, and to determine whether the protected areas meet the feeding requirements for the long-term survival and reproduction of the South China tiger. At the same time, further in-depth research should be conducted on the connectivity of the habitats in the core candidate sites, and the construction of ecological corridors should take place to achieve the true wild release of the South China tiger.

## Figures and Tables

**Table 1:** Information on 8 key prey species of South China tigers.

Family	Genus	Species	English Name	Weight Range (kg)	National Protection Level	Number of Distribution Points
Bovidae	*Capricornis*	*Capricornis milneedwardsi*	Chinese serow	85–140	II	53
	*Naemorhedus*	*Naemorhedus griseus*	Chinese goral	40–50	II	36
Cervidae	*Elaphodus*	*Elaphodus cephalophus*	Tufted deer	15–28	II	95
	*Hydropotes*	*Hydropotes inermis*	Water deer	15–20	II	20
	*Muntiacus*	*Muntiacus reevesi*	Chinese muntjac	9–18	—	69
		*Muntiacus vaginalis*	Red muntjac	20–33	—	102
	*Rusa*	*Rusa unicolor*	Sambar	100–200	II	26
Suidae	*Sus*	*Sus scrofa*	Wild boar	90–200	—	98

**Table 2:** The environmental variables selected for the preliminary model.

Category	Code	Environmental Factors	Resolution	Data Sources
Climatic factors	bio01	Annual mean temperature	30 s	WorldClim (https://worldclim.org/, accessed on 12 July 2023)
	bio02	Mean diural range		
	bio03	Isothermality		
	bio04	Temperature seasonality		
	bio05	Max temperature of warmest month		
	bio06	Min temperature of coldest month		
	bio07	Temperature annual range		
	bio08	Mean temperature of wettest quarter		
	bio09	Mean temperature of driest quarter		
	bio10	Mean temperature of warmest quarter		
	bio11	Mean temperature of coldest quarter		
	bio12	Annual precipitation		
	bio13	Precipitation of wettest month		
	bio14	Precipitation of driest month		
	bio15	Precipitation seasonality		
	bio16	Precipitation of wettest quarter		
	bio17	Precipitation of driest quarter		
	bio18	Precipitation of warmest quarter		
	bio19	Precipitation of coldest quarter		
Terrain factors	altitude	Altitude	90 m	Geospatial Data Cloud (https://www.gscloud.cn/, accessed on 26 July 2023)
	slope	Slope		
	aspect	Aspect		
Water source and vegetation factors	ndvi	Normalized Dierential Vegetation Index	1 km	Resource and Environment Science and Data Center (https://www.resdc.cn/, accessed on 26 July 2023)
	veg	Vegetation type		
	dis_river	Distance from water sources	90 m	Geospatial Data Cloud (https://www.gscloud.cn/, accessed on 26 July 2023)
Human disturbance factors	dis_human	Distance from residential areas	1 km	National Catalogue Service For Geographic Information (https://www.webmap.cn/main.do?method=index, accessed on 3 August 2023)
	dis_road	Distance from road	/	open street map (http://download.geofabrik.de/, accessed on 3 August 2023)

**Table 3:** Changes in habitat area of prey and South China tigers at present, in 2050, and in 2070.

Species	Habitat Suitability	Habitat Area Now (km^2^)	Habitat Area in 2050 (km^2^)	Habitat Area in 2070 (km^2^)	Habitat Change by 2050 (%)	Habitat Change by 2070 (%)
*Capricornis milneedwardsi*	High suitable	54,876	50,138	27,868	−8.63%	−49.21%
	Medium suitable	170,466	179,324	174,466	5.19%	2.34%
	Low suitable	467,156	568,799	526,289	21.75%	12.65%
*Naemorhedus griseus*	High suitable	369,149	170,345	92,282	−53.85%	−75.00%
	Medium suitable	640,326	493,868	397,301	−22.87%	−37.95%
	Low suitable	1,834,674	1,400,069	1,167,314	−23.68%	−36.37%
*Elaphodus cephalophus*	High suitable	213,434	236,695	76,661	10.89%	−64.08%
	Medium suitable	372,400	366,222	306,692	−1.65%	−17.64%
	Low suitable	735,084	738,912	676,538	0.52%	−7.96%
*Hydropotes inermis*	High suitable	1,004,282	958,594	435,657	−4.54%	−56.62%
	Medium suitable	647,448	789,096	1,158,562	21.87%	78.94%
	Low suitable	2,014,916	2,081,083	1,850,296	3.28%	−8.17%
*Muntiacus reevesi*	High suitable	82,783	11,159	5184	−86.52%	−93.73%
	Medium suitable	249,809	58,842	29,950	−76.45%	−88.01%
	Low suitable	606,564	330,524	241,320	−45.51%	−60.21%
*Muntiacus vaginalis*	High suitable	45,199	132,459	120,919	193.05%	167.52%
	Medium suitable	131,419	208,282	189,466	58.48%	44.16%
	Low suitable	479,220	443,652	392,290	−7.42%	−18.13%
*Rusa unicolor*	High suitable	414,874	694,872	770,772	67.48%	85.78%
	Medium suitable	1,074,348	1,518,966	1,760,765	41.38%	63.89%
	Low suitable	1,969,101	2,364,065	2,512,421	20.05%	27.59%
*Sus scrofa*	High suitable	645,192	609,309	522,649	−5.56%	−18.99%
	Medium suitable	1,665,352	1,598,482	1,439,938	−4.01%	−13.53%
	Low suitable	1,715,613	2,010,754	2,047,950	17.20%	19.37%
*Panthera tigris amoyensis*	High suitable	240,184	169,345	85,496	−29.49%	−64.40%
	Medium suitable	962,382	904,320	716,603	−6.03%	−25.53%
	Low suitable	2,176,002	2,665,639	2,829,683	22.50%	30.04%

**Table 4:** The number of protected areas and tigers that can be supported in the core candidate sites.

Patch Number	Patch Area/km^2^	Protected Areas Involved	Number of Tigers
1	10,054.10	Cibagou NNR	23 M, 66 F
2	7518.06	Giant Panda NP, Heizhugou NNR, Mabian Dafengding NNR, Meigu Dafengding NNR, Bayuelin NR, Mamize NR, Maanshan NR	17 M, 49 F
3	7002.50	Giant Panda NP	16 M, 46 F
4	3667.27	/	8 M, 23 F
5	3608.42	/	8 M, 23 F
6	2769.58	Wuyishan NP, Tongbo mountain NR	6 M, 18 F
7	2736.27	Giant Panda NP, Jiuzhaigou NR	6 M, 18 F
8	2619.86	Jiulongshan NNR	5 M, 17 F
9	2311.22	Baima Snow Mountain NNR	5 M, 15 F
10	1995.02	Huaping NNR, Maoershan NNR, Nanshan NP	4 M, 13 F
11	1932.74	Giant Panda NP	4 M, 12 F
12	1433.13	Bamian Mountain NNR, Taoyuandong NNR, Jinggangshan NNR	3 M, 9 F
13	1234.82	/	2 M, 8 F
14	1091.61	Gaoligong Mountain NNR	2 M, 7 F
15	1078.57	Meihuashan NNR	2 M, 7 F
16	1021.09	/	2 M, 6 F
17	1018.29	Yarlung Zangbo Grand Canyon NNR	2 M, 6 F
18	991.45	Hubeishan NNR, Houhe NNR, Mulinzi NNR	2 M, 6 F
19	925.61	Nanling NNR, Mangshan NNR	2 M, 6 F
20	793.57	Qizimei Mountain NNR, Badagongshan NNR	1 M, 5 F

(NNR) National nature reserve; (NP) national park; (NR) nature reserve; (M) male tigers; (F) female tigers.

## Data Availability

The raw data supporting the conclusions of this article will be made available by the authors without undue reservation.

## Supplementary Materials

The following supporting information can be downloaded at: https://www.mdpi.com/article/10.3390/ani14172477/s1, Table S1: prey data of the South China tiger from the Artiodactyla order listed in the Chinese species catalog. Table S2: tiger density statistics. Figure S1: correlation analysis of 27 environmental variables. Figure S2: current and future distribution of suitable habitats for prey. References [52,56,71,72,73,74,75,76] are cited in the supplementary materials.

## References

[1] Mantyka-Pringle C.S., Visconti P., Di Marco M., Martin T.G., Rondinini C., Rhodes J.R. (2015). Climate change modifies risk of global biodiversity loss due to land-cover change. Biol. Conserv..

[2] Pimm S.L. (2008). Biodiversity: Climate Change or Habitat Loss—Which Will Kill More Species?. Curr. Biol..

[3] Koo K.A., Park S.U., Seo C. (2017). Effects of Climate Change on the Climatic Niches of Warm-Adapted Evergreen Plants: Expansion or Contraction?. Forests.

[4] Zhang K., Liu Z., Abdukeyum N., Ling Y. (2022). Potential Geographical Distribution of Medicinal Plant *Ephedra sinica* Stapf under Climate Change. Forests.

[5] Zhang L., Li Q., Kou X., Ouyang Z. (2022). Distributions of two native ungulates at the third pole are highly sensitive to global warming. Glob. Ecol. Conserv..

[6] Ramasamy M., Das B., Ramesh R. (2022). Predicting climate change impacts on potential worldwide distribution of fall armyworm based on CMIP6 projections. J. Pest Sci..

[7] Tang J., Zhang J., Zhao X., Wei W., Hong M., Zhou H., Zhang J., Zhang Z. (2022). The fate of giant panda and its sympatric mammals under future climate change. Biol. Conserv..

[8] Zhou Y., Zhang Z., Zhu B., Cheng X., Yang L., Gao M., Kong R. (2021). MaxEnt Modeling Based on CMIP6 Models to Project Potential Suitable Zones for *Cunninghamia lanceolata* in China. Forests.

[9] Mao C., Ren Q., He C., Qi T. (2023). Assessing direct and indirect impacts of human activities on natural habitats in the Qinghai-Tibet Plateau from 2000 to 2020. Ecol. Indic..

[10] Yang L., Xu H., Pan S., Chen W., Zeng J. (2024). Identifying the impact of global human activities expansion on natural habitats. J. Clean. Prod..

[11] Correa Ayram C.A., Mendoza M.E., Etter A., Pérez Salicrup D.R. (2017). Anthropogenic impact on habitat connectivity: A multidimensional human footprint index evaluated in a highly biodiverse landscape of Mexico. Ecol. Indic..

[12] Roemer G.W., Gompper M.E., Van Valkenburgh B. (2009). The Ecological Role of the Mammalian Mesocarnivore. BioScience.

[13] IUCN The IUCN Red List of Threatened Species. https://www.iucnredlist.org/en.

[14] Tilson R., Defu H., Muntifering J., Nyhus P.J. (2004). Dramatic decline of wild South China tigers *Panthera tigris amoyensis*: Field survey of priority tiger reserves. ORX.

[15] National Forestry and Grassland Administration (NFGA) Biodiversity Conservation in China. https://www.forestry.gov.cn/c/www/szxx/54502.jhtml.

[16] National Forestry and Grassland Administration (NFGA) Leaf Muntjac, Shoudai and Other Wild Animals Were Included in the New ‘Three Haves’ List of Wild Boar and So On. https://www.forestry.gov.cn/search/509801.

[17] Karanth K.U., Stith B.M. (1999). Prey depletion as a critical determinant of tiger population viability. Riding the Tiger: Tiger Conservation in Humman-Dominated Landscapes.

[18] Karanth K.U., Nichols J.D., Kumar N.S., Link W.A., Hines J.E. (2004). Tigers and their prey: Predicting carnivore densities from prey abundance. Proc. Natl. Acad. Sci. USA.

[19] Karanth K.U., Sunquist M.E. (1995). Prey selection by tiger, leopard and dhole in tropical forests. J. Anim. Ecol..

[20] Karanth K.U. (1995). Estimating tiger *Panthera tigris* populations from camera-trap data using capture—Recapture models. Biol. Conserv..

[21] Sanderson E.W., Miquelle D.G., Fisher K., Harihar A., Clark C., Moy J., Potapov P., Robinson N., Royte L., Sampson D. (2023). Range-wide trends in tiger conservation landscapes, 2001–2020. Front. Conserv. Sci..

[22] Qin Y., Nyhus P.J. (2018). Assessing factors influencing a possible South China tiger reintroduction: A survey of international conservation professionals. Environ. Conserv..

[23] The Central People’s Goverment of the People’s Republic of China WWF Positively Evaluated the ‘Chinese Wild Tiger Restoration Plan’. https://www.gov.cn/gzdt/2011-08/04/content_1919539.htm.

[24] The Central People’s Goverment of the People’s Republic of China Forestry Bureau: China Is Preparing the “Chinese Wild Tiger Population Recovery Plan”. https://www.gov.cn/jrzg/2010-10/13/content_1722105.htm.

[25] Tilson R., Nyhus P.J. (2010). Tigers of the World: The Science, Politics and Conservation of Panthera tigris.

[26] Qin Y., Nyhus P.J., Larson C.L., Carroll C.J.W., Muntifering J., Dahmer T.D., Jun L., Tilson R.L. (2015). An assessment of South China tiger reintroduction potential in Hupingshan and Houhe National Nature Reserves, China. Biol. Conserv..

[27] Tang T., Li J., Sun H., Velázquez J. (2023). Integrated approach considering seasonal variability and habitat uncertainty to map habitat for the prey of South China tiger. Ecol. Indic..

[28] Tang T., Li J., Sun H., Deng C. (2021). Priority areas identified through spatial habitat suitability index and network analysis: Wild boar populations as proxies for tigers in and around the Hupingshan and Houhe National Nature Reserves. Sci. Total Environ..

[29] Wang X., He J., Mou X., Zhu Z., Chai H., Liu G., Rao S., Zhang X. (2021). 20 Years of Ecological Protection and Restoration in China: Review and Prospect. Chin. J. Environ. Manag..

[30] Yuan X., Chen W., Lu K., Lu Y., Zhang S. (1994). Investigation on South China Tigers and Their Habitats in Guangdong Province. Chin. J. Wildl..

[31] Hayward M.W., Jędrzejewski W., Jêdrzejewska B. (2012). Prey preference of the tiger *Panthera tigris*. J. Zool..

[32] Tilson R., Traylor-Holzer K., Jiang Q.M. (1997). The Decline and Impending Extinction of the South China Tiger.

[33] Liang J., Ding Z., Jiang Z., Yang X., Xiao R., Singh P.B., Hu Y., Guo K., Zhang Z., Hu H. (2021). Climate change, habitat connectivity, and conservation gaps: A case study of four ungulate species endemic to the Tibetan Plateau. Landsc. Ecol..

[34] Malakoutikhah S., Fakheran S., Hemami M., Tarkesh M., Senn J. (2020). Assessing future distribution, suitability of corridors and efficiency of protected areas to conserve vulnerable ungulates under climate change. Divers. Distrib..

[35] Meng B., Huang X., Xie B., Wang W., Huang J., Zhang T., Ran J., Zhang M. (2023). Distribution of suitable habitat for ungulates in Fanjingshan National Nature Reserve Reserve, Guizhou Province. Acta Theriol. Sin..

[36] Zhang K., Zhang Y., Tao J. (2019). Predicting the Potential Distribution of *Paeonia veitchii* (Paeoniaceae) in China by Incorporating Climate Change into a Maxent Model. Forests.

[37] Phillips S.J., Anderson R.P., Schapire R.E. (2006). Maximum entropy modeling of species geographic distributions. Ecol. Model..

[38] Cobos M.E., Peterson A.T., Barve N., Osorio-Olvera L. (2019). kuenm: An R package for detailed development of ecological niche models using Maxent. PeerJ.

[39] Muscarella R., Galante P.J., Soley-Guardia M., Boria R.A., Kass J.M., Uriarte M., Anderson R.P. (2014). ENMeval: An R package for conducting spatially independent evaluations and estimating optimal model complexity for Maxent ecological niche models. Methods Ecol. Evol..

[40] Fielding A.H., Bell J.F. (1997). A review of methods for the assessment of prediction errors in conservation presence/absence models. Environ. Conserv..

[41] Shcheglovitova M., Anderson R.P. (2013). Estimating optimal complexity for ecological niche models: A jackknife approach for species with small sample sizes. Ecol. Model..

[42] Swets J.A. (1988). Measuring the Accuracy of Diagnostic Systems. Science.

[43] Mu H., Li X., Wen Y., Huang J., Du P., Su W., Miao S., Geng M. (2022). A global record of annual terrestrial Human Footprint dataset from 2000 to 2018. Sci. Data.

[44] O’Brien T.G., Kinnaird M.F., Wibisono H.T. (2003). Crouching tigers, hidden prey: Sumatran tiger and prey populations in a tropical forest landscape. Anim. Conserv..

[45] Sunquist M. (2010). Chapter2-What Is a Tiger? Ecology and Behavior. Tigers of the World: The Science, Politics and Conservation of Panthera tigris.

[46] Zeng J., Hu J., Shi Y., Li Y., Guo Z., Wang S., Song S. (2022). Effects of Climate Change on the Habitat of the Leopard (*Panthera pardus*) in the Liupanshan National Nature Reserve of China. Animals.

[47] Li W., Shi M., Huang Y., Chen K., Sun H., Chen J. (2019). Climatic Change Can Influence Species Diversity Patterns and Potential Habitats of Salicaceae Plants in China. Forests.

[48] Markov N., Pankova N., Morelle K. (2019). Where winter rules: Modeling wild boar distribution in its north-eastern range. Sci. Total Environ..

[49] Awasthi N., Kumar U., Qureshi Q., Pradhan A., Chauhan J.S., Jhala Y.V. (2016). Effect of human use, season and habitat on ungulate density in Kanha Tiger Reserve, Madhya Pradesh, India. Reg. Environ. Change.

[50] Biswas S., Kumar S., Bandhopadhyay M., Patel S.K., Lyngdoh S., Pandav B., Mondol S. (2023). What drives prey selection? Assessment of Tiger (*Panthera tigris*) food habits across the Terai-Arc Landscape, India. MAMM.

[51] Dahal B.R., Amin R., Lamichhane B.R., Giri S.R., Acharya H., Acharya H.R., Harihar A. (2023). Setting recovery targets for a charismatic species in an iconic protected area complex: The case of tigers (*Panthera tigris*) in Chitwan–Parsa National Parks, Nepal. Conserv. Sci. Pract..

[52] Qi J., Gu J., Ning Y., Miquelle D.G., Holyoak M., Wen D., Liang X., Liu S., Roberts N.J., Yang E. (2021). Integrated assessments call for establishing a sustainable meta-population of Amur tigers in northeast Asia. Biol. Conserv..

[53] Mehlich R. (2005). A Preliminary Habitat Suitability Analysis for the Restoration of South China Tigers in the Hupingshan Reserve, China. Atlas Maine.

[54] Letro L., Fischer K., Duba D., Tandin T. (2022). Occupancy patterns of prey species in a biological corridor and inferences for tiger population connectivity between national parks in Bhutan. Oryx.

[55] Penjor U., Kaszta Ż., Macdonald D.W., Cushman S.A. (2021). Prioritizing areas for conservation outside the existing protected area network in Bhutan: The use of multi-species, multi-scale habitat suitability models. Landsc. Ecol..

[56] Thinley P., Rajaratnam R., Morreale S.J., Lassoie J.P. (2021). Assessing the adequacy of a protected area network in conserving a wide-ranging apex predator: The case for tiger (*Panthera tigris*) conservation in Bhutan. Conserv. Sci. Pract..

[57] Gray T.N.E., Crouthers R., Ramesh K., Vattakaven J., Borah J., Pasha M.K.S., Lim T., Phan C., Singh R., Long B. (2017). A framework for assessing readiness for tiger *Panthera tigris* reintroduction: A case study from eastern Cambodia. Biodivers. Conserv..

[58] Jhala Y., Gopal R., Mathur V., Ghosh P., Negi H.S., Narain S., Yadav S.P., Malik A., Garawad R., Qureshi Q. (2021). Recovery of tigers in India: Critical introspection and potential lessons. People Nat..

[59] Belote R.T., Wilson M.B. (2020). Delineating greater ecosystems around protected areas to guide conservation. Conserv. Sci. Pract..

[60] Dinerstein E., Joshi A.R., Vynne C., Lee A.T.L., Pharand-Deschênes F., França M., Fernando S., Birch T., Burkart K., Asner G.P. (2020). A “Global Safety Net” to reverse biodiversity loss and stabilize earth’s climate. Sci. Adv..

[61] Dinerstein E., Loucks C., Wikramanayake E., Ginsberg J., Sanderson E., Seidensticker J., Forrest J., Bryja G., Heydlauff A., Klenzendorf S. (2007). The Fate of Wild Tigers. BioScience.

[62] Kenney J.S., Smith J.L.D., Starfield A.M., Mcdougal C.W. (1995). The Long-Term Effects of Tiger Poaching on Population Viability. Conserv. Biol..

[63] Linkie M., Chapron G., Martyr D.J., Holden J., Leader-Williams N. (2006). Assessing the viability of tiger subpopulations in a fragmented landscape. J. Appl. Ecol..

[64] Carter N., Killion A., Easter T., Brandt J., Ford A. (2020). Road development in Asia: Assessing the range-wide risks to tigers. Sci. Adv..

[65] Dertien J.S., Negi H., Dinerstein E., Krishnamurthy R., Negi H.S., Gopal R., Gulick S., Pathak S.K., Kapoor M., Yadav P. (2023). Mitigating human-wildlife conflict and monitoring endangered tigers using a real-time camera-based alert system. BioScience.

[66] Rathore C.S., Dubey Y., Shrivastava A., Pathak P., Patil V. (2012). Opportunities of Habitat Connectivity for Tiger (*Panthera tigris*) between Kanha and Pench National Parks in Madhya Pradesh, India. PLoS ONE.

[67] Wen D., Qi J., Long Z., Gu J., Tian Y., Roberts N.J., Yang E., Kong W., Zhao Y., Sun Q. (2022). Conservation potentials and limitations of large carnivores in protected areas: A case study in Northeast China. Conserv. Sci. Pract..

[68] Fàbregas M.C., Fosgate G.T., Koehler G.M. (2015). Hunting performance of captive-born South China tigers (*Panthera tigris amoyensis*) on free-ranging prey and implications for their reintroduction. Biol. Conserv..

[69] Wang C., Wu D.-D., Yuan Y.-H., Yao M.-C., Han J.-L., Wu Y.-J., Shan F., Li W.-P., Zhai J.-Q., Huang M. (2023). Population genomic analysis provides evidence of the past success and future potential of South China tiger captive conservation. BMC Biol..

[70] Ning Y., Liu D., Gu J., Zhang Y., Roberts N.J., Guskov V.Y., Sun J., Liu D., Gong M., Qi J. (2024). The genetic status and rescue measure for a geographically isolated population of Amur tigers. Sci. Rep..

[71] Sarkar M.S., Ramesh K., Johnson J.A., Sen S., Nigam P., Gupta S.K., Murthy R.S., Saha G.K. (2016). Movement and home range characteristics of reintroduced tiger (*Panthera tigris*) population in Panna Tiger Reserve, central India. Eur. J. Wildl. Res..

[72] Chundawat R.S., Sharma K., Gogate N., Malik P.K., Vanak A.T. (2016). Size matters: Scale mismatch between space use patterns of tigers and protected area size in a Tropical Dry Forest. Biol. Conserv..

[73] Dendup P., Lham C., Wangchuk W., Jamtsho Y. (2023). Tiger abundance and ecology in Jigme Dorji National Park, Bhutan. Glob. Ecol. Conserv..

[74] Duangchantrasiri S., Umponjan M., Simcharoen S., Pattanavibool A., Chaiwattana S., Maneerat S., Kumar N.S., Jathanna D., Srivathsa A., Karanth K.U. (2016). Dynamics of a low-density tiger population in Southeast Asia in the context of improved law enforcement. Conserv. Biol..

[75] Phumanee W., Steinmetz R., Phoonjampa R., Weingdow S., Phokamanee S., Bhumpakphan N., Savini T. (2021). Tiger density, movements, and immigration outside of a tiger source site in Thailand. Conserv. Sci. Pract..

[76] Sarkar M.S., Niyogi R., Masih R.L., Hazra P., Maiorano L., John R. (2021). Long-distance dispersal and home range establishment by a female sub-adult tiger (*Panthera tigris*) in the Panna landscape, central India. Eur. J. Wildl. Res..

