# Supplementary material for: Predicting the Impact of Climate Change on the Selection of Reintroduction Sites for the South China Tiger (Panthera tigris amoyensis) in China
Source: Animals (Basel). 2024 Aug 26;14(17):2477. doi: 10.3390/ani14172477 (PMC11394341; doi:10.3390/ani14172477)
Supplement: Supplementary file 1 [file animals-14-02477-s001.zip › animals-3119457-supplementary.pdf]

Supplementary Materials

Table S1. Prey data of the South China tiger from the Artiodactyla order listed in the Chinese species catalog.

Family	Genus	Species	English name	Activity range	Weight range（kg）	National protection level
Bovidae	*Bos*	*Bos gaurus*	Indian Bison	Yunnan, Tibet	650-800	I
Bovidae	*Bos*	*Bos mutus*	Wild Yak	Gansu, Qinghai, Tibet, Xinjiang	500-600	I
Bovidae	*Budorcas*	*Budorcas taxicolor*	Himalayan Takin	Yunnan, Tibet	200-300	I
Bovidae	*Budorcas*	*Budorcas tibetana*	Chinese Takin	Gansu, Sichuan, Hubei, Shaanxi	250-500	I
Bovidae	*Capra*	*Capra sibirica*	AsiaticIbex	Gansu, Inner Mongolia, Shandong, Xinjiang	40-120	II
Bovidae	*Capricornis*	*Capricornis milneedwardsii*	Chinese Serow	Gansu, Guangdong, Anhui, Jiangxi, Yunnan, Shaanxi, Tibet, Guizhou, Fujian, Sichuan, Hubei, Huadong, Zhejiang, Guangxi, Chongqing, Qinghai	85-140	II
Bovidae	*Capricornis*	*Capricornis rubidus*	Red Serow	Yunnan	50-140	II
Bovidae	*Capricornis*	*Capricornis swinhoei*	Taiwan Serow	Taiwan	17-25	I
Bovidae	*Capricornis*	*Capricornis thar*	Himalayan Serow	Tibet	60-90	I
Bovidae	*Gazella*	*Gazella subgutturosa*	Goitered Gazelle	Gansu, Inner Mongolia, Qinghai, Shaanxi, Xinjiang	29-42	II
Bovidae	*Hemitragus*	*Hemitragus jemlahicus*	Himalayan Tahr	Tibet	30-90	I
Bovidae	*Naemorhedus*	*Naemorhedus baileyi*	Red Goral	Yunnan, Tibet	20-30	I
Bovidae	*Naemorhedus*	*Naemorhedus caudatus*	Chinese Gray Goral	Jilin, Heilongjiang	32-42	II
Bovidae	*Naemorhedus*	*Naemorhedus evansi*	Burmese Goral	Yunnan	20-40	II
Bovidae	*Naemorhedus*	*Naemorhedus goral*	Himalayan Goral	Tibet	35-42	I
Bovidae	*Naemorhedus*	*Naemorhedus griseus*	Chinese Goral	Gansu, Guangdong, Hunan, Inner Mongolia, Anhui, Jiangxi, Yunnan, Shaanxi, Tibet, Guizhou, Fujian, Hebei, Sichuan, Hubei, Huadong, Zhejiang, Guangxi, Chongqing, Henan, Shanxi	40-50	II

Family	Genus	Species	English name	Activity range	Weight range （kg）	National protection level
Bovidae	*Ovis*	*Ovis ammon*	Wild Sheep	Gansu, Sichuan, Inner Mongolia, Huabei, Qinghai, Tibet, Xinjiang	65-185	II
Bovidae	*Pantholops*	*Pantholops hodgsonii*	Chiru	Qinghai, Tibet, Xinjiang	45-60	I
Bovidae	*Procapra*	*Procapra gutturosa*	Mongolian Gazelle	Gansu, Inner Mongolia	20-39	I
Bovidae	*Procapra*	*Procapra picticaudata*	Tibetan Gazelle	Gansu, Sichuan, Qinghai, Tibet, Xinjiang	11-16	II
Bovidae	*Procapra*	*Procapra przewalskii*	Przewalski's Gazelle	Qinghai	17-32	I
Bovidae	*Pseudois*	*Pseudois nayaur*	Bharal	Gansu, Sichuan, Inner Mongolia, Yunnan, Qinghai, Tibet, Ningxia, Xinjiang	60-75	II
Camelidae	*Camelus*	*Camelus ferus*	Bactrian Camel	Gansu, Inner Mongolia, Qinghai, Xinjiang	450-600	I
Cervidae	*Alces*	*Alces alces*	Eurasian Elk	Inner Mongolia, Heilongjiang, Xinjiang	400-600	I
Cervidae	*Capreolus*	*Capreolus pygargus*	Siberian Roe Deer	Gansu, Liaoning, Inner Mongolia, Huabei, Shaanxi, Ningxia, Xinjiang, Hebei, Sichuan, Hubei, Jilin, Henan, Heilongjiang, Shanxi, Qinghai	30-50	—
Cervidae	*Cervus*	*Cervus elaphus*	Red Deer	Gansu, Sichuan, Inner Mongolia, Jilin, Heilongjiang, Qinghai, Tibet, Ningxia, Hebei, Xinjiang	150-200	II
Cervidae	*Cervus*	*Cervus nippon*	Sika Deer	Gansu, Sichuan, Hubei, Huadong, Jilin, Zhejiang, Taiwan, Jiangxi, Huabei, Shanxi	70-100	I
Cervidae	*Elaphodus*	*Elaphodus cephalophus*	Tufted Deer	Gansu, Guangdong, Hunan, Jiangxi, Yunnan, Shaanxi, Tibet, Guizhou, Fujian, Sichuan, Hubei, Huadong, Zhejiang, Guangxi, Chongqing, Qinghai	15-28	II
Cervidae	*Elaphurus*	*Elaphurus davidianus*	Pere David's Deer	Beijing, South China sea, Hunan, Hubei, Jiangxi, Jiangsu, Hebei	120-180	I
Cervidae	*Hydropotes*	*Hydropotes inermis*	Water Deer	Liaoning, Shanghai, Zhejiang, Jilin, Anhui, Jiangxi, Jiangsu	15-20	II
Cervidae	*Muntiacus*	*Muntiacus crinifrons*	Black Muntjac	Zhejiang, Anhui, Jiangxi, Fujian	21-30	I
Cervidae	*Muntiacus*	*Muntiacus feae*	Fea's Muntjac	Yunnan, Tibet	15-25	—
Cervidae	*Muntiacus*	*Muntiacus gongshanen*	Gongshan Muntjac	Yunnan, Tibet	16-24	II

Family	Genus	Species	English name	Activity range	Weight range（kg）	National protection level
		sis				
Cervidae	*Muntiacus*	*Muntiacus reevesi*	Chinese Muntjac	Guangdong, Gansu, Hunan, Jiangxi, Yunnan, Shaanxi, Guizhou, Ningxia, Huadong, Guangxi, Zhejiang, Taiwan, Chongqing	9-18	—
Cervidae	*Muntiacus*	*Muntiacus vaginalis*	Red Muntjac	Sichuan, Guangxi, Hongkong, Yunnan, Tibet, Huanan, Guizhou, Hainan	20-33	—
Cervidae	*Przewalskium*	*Przewalskium albirostris*	Thorold's Deer	Gansu, Sichuan, Yunnan, Qinghai, Tibet	130-200	I
Cervidae	*Rucervus*	*Rucervus eldii*	Eld's Deer	Hainan	60-100	I
Cervidae	*Rusa*	*Rusa unicolor*	Sambar	Guangdong, Sichuan, Hunan, Guangxi, Taiwan, Chongqing, Jiangxi, Yunnan, Guizhou, Hainan	100-200	II
Moschidae	*Moschus*	*Moschus anhuiensis*	Anhui Musk Deer	Hubei, Anhui, Henan	7-9	I
Moschidae	*Moschus*	*Moschus berezovskii*	Chinese Forest Musk Deer	Guangdong, Gansu, Hunan, Jiangxi, Yunnan, Shaanxi, Tibet, Guizhou, Ningxia, Sichuan, Hubei, Guangxi, Henan, Qinghai	6-9	I
Moschidae	*Moschus*	*Moschus chrysogaster*	Alpine Musk Deer	Gansu, Sichuan, Yunnan, Qinghai, Tibet, Ningxia	10-15	I
Moschidae	*Moschus*	*Moschus fuscus*	Black Musk Deer	Yunnan, Tibet	7-9	I
Moschidae	*Moschus*	*Moschus leucogaster*	Himalayan Musk Deer	Tibet	11-16	I
Moschidae	*Moschus*	*Moschus moschiferus*	Siberian Musk Deer	Beijing, Liaoning,Inner Mongolia, Jilin, Henan, Heilongjiang, Shanxi, Xinjiang, Hebei	9-13	I
Suidae	*Sus*	*Sus scrofa*	Wild Boar	Gansu, Anhui, Jiangxi, Shaanxi, Tibet, Fujian, Hainan, Tianjin, Xinjiang, Sichuan, Hubei, Zhejiang, Jilin, Chongqing, Shanxi, Heilongjiang, Guangdong, Liaoning, Hunan, Inner Mongolia, Yunnan, Ningxia, Guizhou, Hebei, Guangxi, Taiwan, Jiangsu, Qinghai	90-200	—
Tragulidae	*Tragulus*	*Tragulus kanchil*	Lesser Mouse Deer	Yunnan	1-3	—

Table S2. Tiger density statistics

Tiger	Density（/100 km²）	Location	Cite
Male Tiger	0.75	Panna Tiger Reserve, central India	[1]
	0.55	Panna Tiger Reserve, central India	[2]
	0.66	Jigme Dorji National Park, Bhutan	[3]
	0.65	Laoyeling, Zhangguangcailing (ZGCL), Wanda Mountains (WDM) and Lesser Khingan Mountains (LKM)	[4]
	1.25-2.01	Huai Kha Khaeng Wildlife Sanctuary, western Thailand	[5]
	0.36	Mae Wong National Park and Khlong Lan National Park, western Thailand	[6]
	0.46	The Eastern Himalayan Kingdom of Bhutan	[7]
Female Tiger	1.35	Panna Tiger Reserve, central India	[1]
	2.41	Panna Tiger reserve and Ranipur Wildlife Sanctuary, central India	[8]
	2.14	Panna Tiger Reserve, central India	[2]
	1.11	Jigme Dorji National Park, Bhutan	[3]

References

1. Sarkar, M.S.; Ramesh, K.; Johnson, J.A.; Sen, S.; Nigam, P.; Gupta, S.K.; Murthy, R.S.; Saha, G.K. Bhutan. Movement and home range characteristics of reintroduced tiger (*Panthera tigris*) population in Panna Tiger Reserve, central India. *Eur J Wildl Res* **2016**, *62*, 537–547.
2. Chundawat, R.S.; Sharma, K.; Gogate, N.; Malik, P.K.; Vanak, A.T. Size matters: Scale mismatch between space use patterns of tigers and protected area size in a Tropical Dry Forest. *Biological Conservation* **2016**, *197*, 146–153.
3. Dendup, P.; Lham, C.; Wangchuk, W.; Jamtsho, Y. Tiger abundance and ecology in Jigme Dorji National Park, Bhutan. *Global Ecology and Conservation* **2023**, *42*, e02378.
4. Qi, J.; Gu, J.; Ning, Y.; Miquelle, D.G.; Holyoak, M.; Wen, D.; Liang, X.; Liu, S.; Roberts, N.J.; Yang, E.; et al. Integrated assessments call for establishing a sustainable meta-population of Amur tigers in northeast Asia. *Biological Conservation* **2021**, *261*, 109250.
5. Duangchantrasiri, S.; Umponjan, M.; Simcharoen, S.; Pattanavibool, A.; Chaiwattana, S.; Maneerat, S.; Kumar, N.S.; Jathanna, D.; Srivathsa, A.; Karanth, K.U. Dynamics of a low-density tiger population in Southeast Asia in the context of improved law enforcement. *Conserv. Biol.* **2016**, *30*, 639–648.
6. Phumanee, W.; Steinmetz, R.; Phoonjampa, R.; Weingdow, S.; Phokamanee, S.; Bhumpakphan, N.; Savini, T. Tiger density, movements, and immigration outside of a tiger source site in Thailand. *Conservation Science and Practice* **2021**, *3*, e560.
7. Thinley, P.; Rajaratnam, R.; Morreale, S.J.; Lassoie, J.P. Assessing the adequacy of a protected area network in conserving a wide-ranging apex predator: The case for tiger (*Panthera tigris*) conservation in Bhutan. *Conservation Science and Practice* **2021**, *3*, e318.
8. Sarkar, M.S.; Niyogi, R.; Masih, R.L.; Hazra, P.; Maiorano, L.; John, R. Long-distance dispersal and home range establishment by a female sub-adult tiger (*Panthera tigris*) in the Panna landscape, central India. *Eur J Wildl Res* **2021**, *67*, 54.

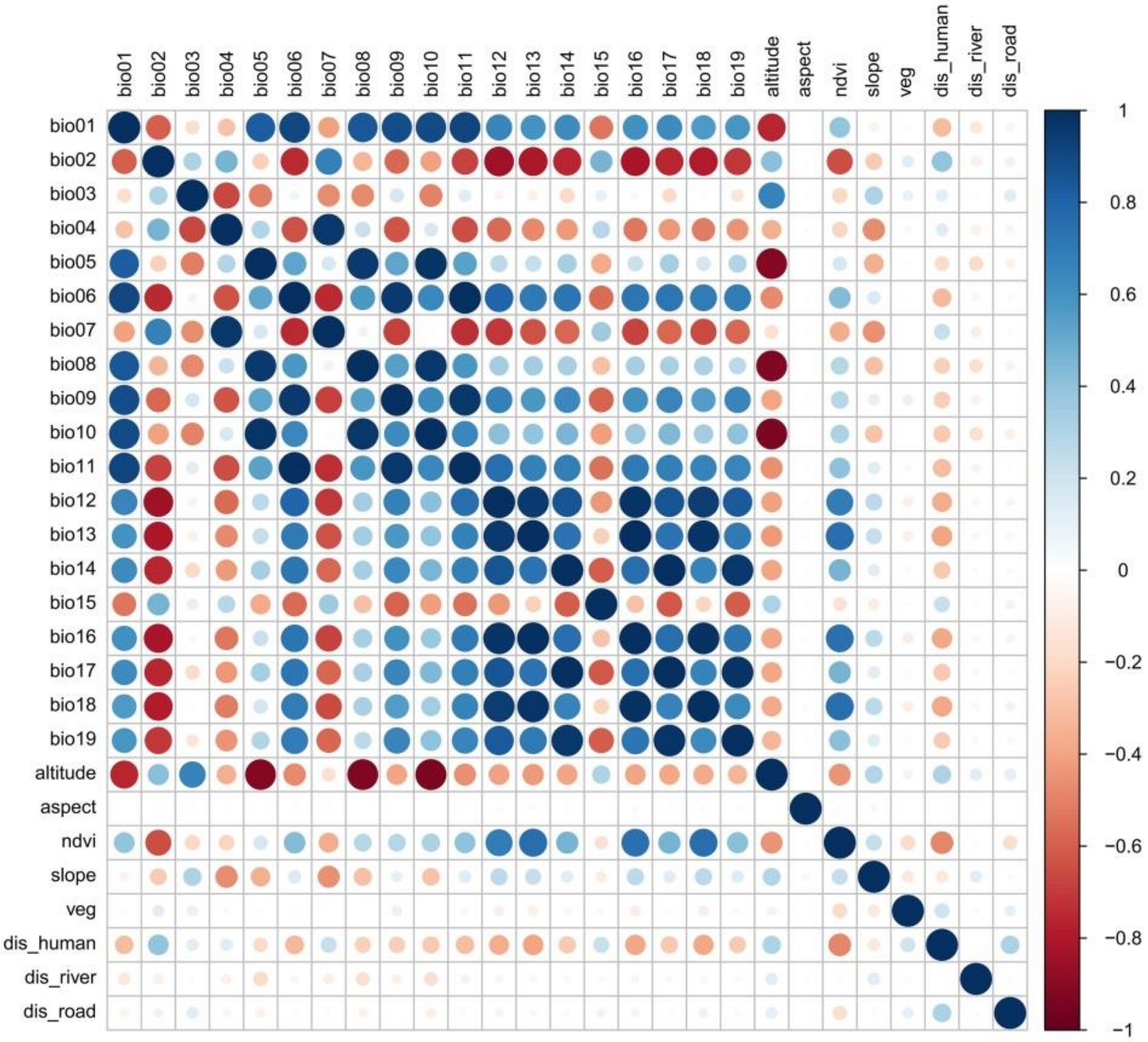

Figure S1. Correlation analysis of 27 environmental variables

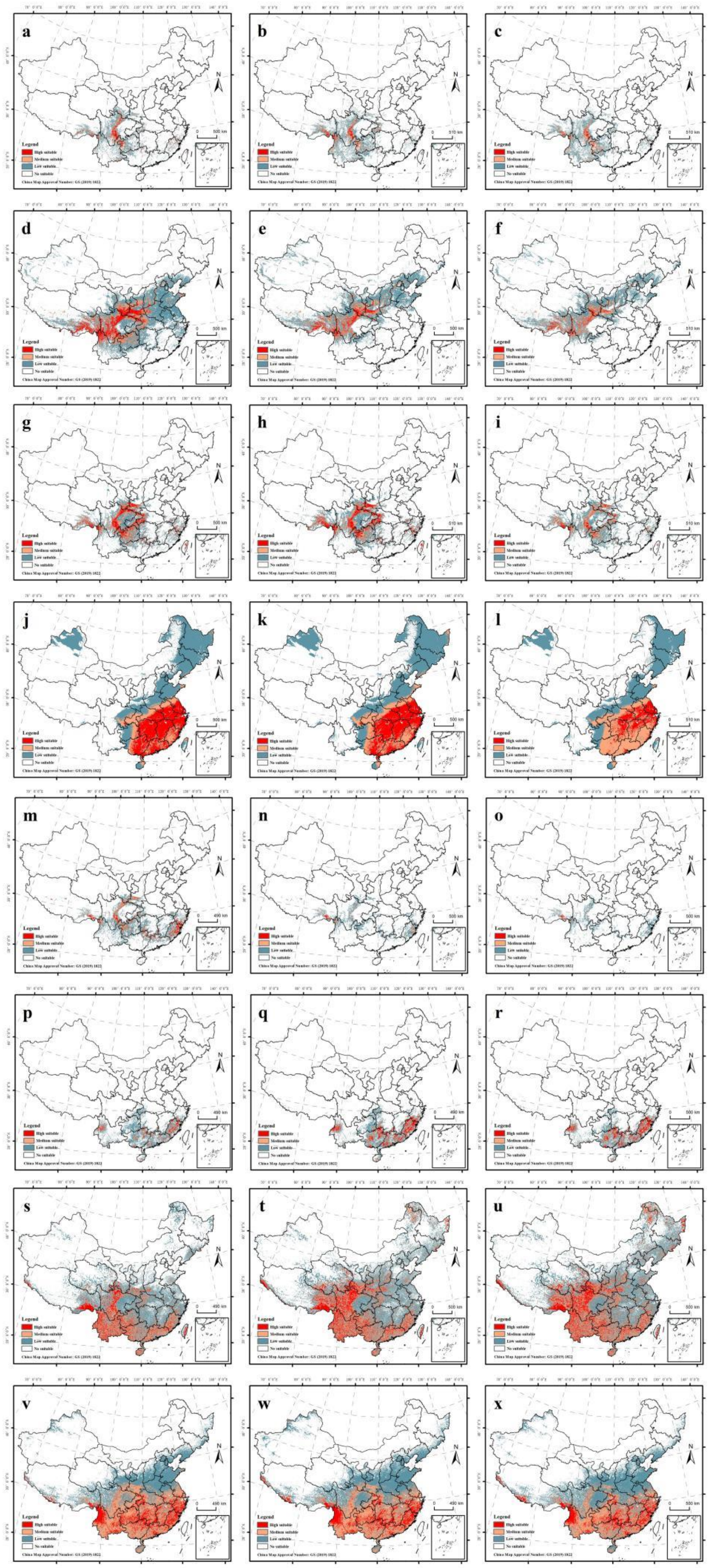

a
b
c
d
e
f
g
h
i
j
k
l
m
n
o
p
q
r
s
t
u
v
w
x
Legend
High suitable
Medium suitable
Low suitable
No suitable
China Map Approval Number: GS (2019) 1822

Figure S2. Current and future distribution of suitable habitats for prey

(a-c are distribution maps of the suitable habitats for the Chinese serow currently, in 2050, and in 2070; d-f are distribution maps of the suitable habitats for the Chinese goral currently, in 2050, and in 2070; g-i are distribution maps of the suitable habitats for the Tufted deer currently, in 2050, and in 2070; j-l are distribution maps of the suitable habitats for the Water deer currently, in 2050, and in 2070; m-o are distribution maps of the suitable habitats for the Chinese muntjac currently, in 2050, and in 2070; p-r are distribution maps of the suitable habitats for the Red muntjac currently, in 2050, and in 2070; s-u are distribution maps of the suitable habitats for the Sambar currently, in 2050, and in 2070; v-x are distribution maps of the suitable habitats for the Wild boar currently, in 2050, and in 2070)
